# Advances in Contraceptive Vaccine Development: A Comprehensive Review

**DOI:** 10.3390/vaccines13070692

**Published:** 2025-06-26

**Authors:** Wen Gao, Xiaoting Shen, Peipei Li, Chanchan Xiao, Yongxia Wang

**Affiliations:** 1 NHC Key Laboratory of Male Reproduction and Genetics, Guangdong Provincial Reproductive Science Institute (Guangdong Provincial Fertility Hospital), Guangzhou 510600, China; gaowen9101@163.com (W.G.); shenxt@gdszjk.org.cn (X.S.); lipp@gdszjk.org.cn (P.L.); 2 The Affiliated Hospital of Xiangnan University, Chenzhou 423000, China; 3 Institute of Geriatric Immunology, School of Medicine, Jinan University, Guangzhou 510632, China

**Keywords:** contraceptive vaccines, immunocontraception, sperm production, sperm antigens, zona pellucida of oocytes, gamete outcomes

## Abstract

The issues of uncontrolled global population growth and unintended pregnancies are severe, and the existing contraceptive methods have numerous limitations, making the development of novel contraceptive technologies urgent. Contraceptive vaccines offer a promising alternative to traditional contraception methods. This article reviews the three developmental stages of contraceptive vaccines, including early exploration, technical bottlenecks, and innovative development directions in the new era. This article also summarizes the targets of immunocontraception, covering the current research status of contraceptive vaccines targeting sperm production, sperm antigens, oocyte zona pellucida, and gamete outcomes. Furthermore, this article explores the advantages of contraceptive vaccines in terms of efficiency, non-invasiveness, reversibility, and the promotion of gender equality. Challenges associated with clinical translation and real-world implementation are also critically analyzed.

## 1. Global Demand for Contraception and Public Health

The uncontrolled population growth and unintended pregnancies remain pressing global social and health issues. According to the World Health Statistics report, among the 1.9 billion women of reproductive age group (15–49 years) worldwide in 2021, 1.1 billion have a need for family planning; of these, 874 million are using modern contraceptive methods, and 164 million have an unmet need for contraception [1,2]. The proportion of the need for family planning satisfied by modern methods has stagnated globally at around 77% from 2015 to 2022 but increased from 52% to 58% in sub-Saharan Africa [3].

Inadequate contraceptive use is one of the key contributing factors to unintended pregnancies and induced abortions. First, unsafe abortion remains a major cause of maternal mortality and severe complications (e.g., intrauterine adhesions) worldwide. It not only leads to individual health tragedies but also consumes substantial, otherwise preventable, medical resources and societal costs. About 287,000 maternal deaths, almost 95% occurring in low-to-lower-middle income countries, were recorded during and after pregnancy in 2020 [4]. According to statistics, public hospitals in Nigeria’s Ogun and Lagos States, as well as the Federal Capital Territory, spend approximately USD 807,442 annually on treating abortion-related complications [5]. Second, unintended pregnancy exacerbates socioeconomic inequalities. Unintended pregnancies also perpetuate a vicious cycle of poverty, denied educational opportunities, and gender inequality. Poor women in remote areas are the least likely to receive adequate health care [6]. The consequences of unintended pregnancy—whether resulting in forced childbirth or unsafe abortion—may compel women to discontinue education or professional careers, trapping them in cycles of poverty and restricting opportunities for social participation and upward mobility [7]. This phenomenon perpetuates intergenerational poverty. Therefore, improving access to safe, effective, affordable, and acceptable contraceptive methods is not only a critical public health intervention but also a fundamental strategy for promoting gender equality, reducing poverty, safeguarding reproductive rights, enhancing population health, and ultimately advancing social equity and sustainable development.

However, significant gaps exist in the accessibility, acceptability, and safety of current contraceptive methods. Modern contraceptive options available to women include tubal ligation, barrier methods, hormonal methods (pills, implants, injectables, progesterone intrauterine devices (IUDs), or emergency contraception), and non-hormonal IUDs [8]. Although hormonal contraceptives can be 99% effective, approximately 30% of users discontinue them within a year due to side effects like headaches, mood swings, and thrombotic risks. The IUD offers long-term and highly effective contraception, but its insertion can cause pain, bleeding, or uterine perforation (with an incidence rate of approximately 1/1000), and about 20% of health care facilities in lower-income countries lack professional training for this procedure. Men generally use two contraceptive methods: vasectomy and condoms. While condoms are the only contraceptive method that can prevent sexually transmitted infections (STIs), their typical use failure rate is as high as 18% [9]. Vasectomy is associated with high surgical costs, uncertain success rates of vasovasostomy reversal, and a non-negligible incidence of postoperative complications [10]. Classical hormonal male contraceptives have demonstrated systemic side effects in clinical reports, along with concerns regarding failure rates and spermatogenesis recovery rates [11,12]. Novel hormonal agents such as GnRH antagonists require daily or weekly injections, incurring prohibitive costs [13], rendering them suboptimal contraceptive choices. Non-hormonal male contraceptives (e.g., HC-056456 [14] and YCT529 [15]) still require long-term clinical trials to validate their efficacy and reversibility. Herbal contraceptives like *Justicia gendarussa* Burm f. are empirically used as male contraceptives in endemic regions [16]. However, their complex phytochemical composition, undefined toxicity profiles, and the current lack of systematic preclinical and clinical investigations limit their applicability. These limitations underscore the urgent global need for innovative contraceptive technologies. Therefore, finding a safe, convenient, effective, and reversible contraceptive measure is profoundly significant in controlling population growth and avoiding unintended pregnancies.

Immunocontraception represents a fertility control strategy rooted in immunological principles. Its core lies in the selection of key antigens associated with the reproductive process, the elimination of non-dominant antigen epitopes such as inhibitory epitopes and autoantigen cross-reactive epitopes, and the incorporation of T cell epitopes that promote immune responses in the body. These modified antigens are then coupled with appropriate carriers to create contraceptive vaccines. These vaccines induce corresponding humoral or cellular immune responses in the body, ultimately achieving the goal of preventing conception [17,18]. This review provides a comprehensive overview of contraceptive vaccines, covering their development history, target antigens and current research progress, and comparative advantages versus associated challenges.

## 2. The Development of Contraceptive Vaccines

### 2.1. Early Exploration Phase (1900s–1980s): Concept Validation from Animal Models to Human Trials

As early as 1899, Nobel laureates Landsteiner and Metchnikoff independently demonstrated that the injection of heterologous spermatozoa could elicit an antibody response in animals [19]. Subsequently, in 1937, surgeon Morris applied for a U.S. patent for an anti-sperm contraceptive vaccine. Although this vaccine showed reversible contraceptive effects in women of reproductive age, its unclear side effects hindered clinical application [20]. The anti-human chorionic gonadotropin (anti-hCG) vaccine was the first and only contraceptive vaccine to successfully complete Phase II efficacy trials. The reversibility and safety of anti-fertility vaccines, specifically anti-hCG vaccines, were established in Phase I trials conducted at the National Institute of Immunology in New Delhi, India. However, its limited duration of antibody persistence (only 2–3 months) precluded widespread adoption [21].

### 2.2. Technological Bottleneck Phase (1990s–2010s): Antigen Design Optimization

**Figure 1:**
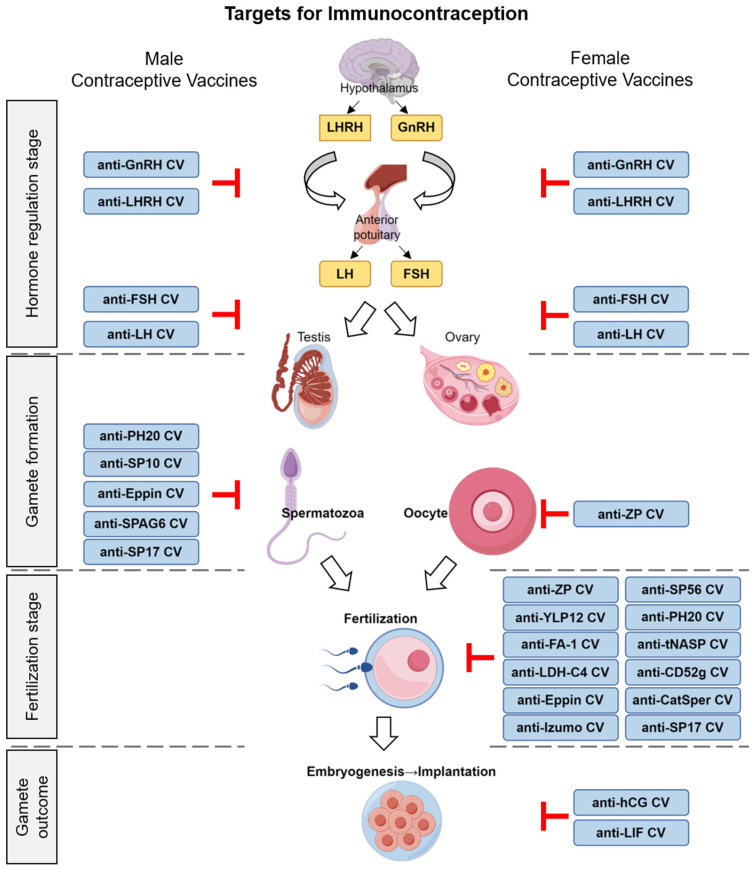
Schematic of early human reproductive processes and immunocontraceptive targets for vaccine design.

The technology of recombinant proteins matured and rapidly advanced during this phase. Researchers designed and developed various anti-sperm, anti-zona pellucida, and anti-reproductive hormone vaccines with different antigenic epitopes using genetic engineering techniques (Figure 1). With innovations in vaccine development, peptide vaccines (e.g., ZP3 peptide vaccine) [22] and DNA vaccines (e.g., pCMV4-rZPC’ DNA vaccine) [23] were also applied to contraceptive vaccine research.

### 2.3. New Era (2020s–Present): Delivery System Innovation and Precision Design

The remarkable success of COVID-19 mRNA vaccines during the pandemic has significantly propelled the development of mRNA vaccine technology in other applications. Recently, a vaginal film formulated with the anti-CD52g monoclonal antibody HCA was tested in women in a Phase I clinical trial [24], following initial studies [25]. Alternative antibody delivery systems, such as intravaginal rings and topical mRNA applications, are being explored [9].

With advancements in structural biology, structure-based antigen vaccine design has emerged. In June 2016, two research teams from Canada and Japan independently discovered that the sperm surface protein IZUMO1 and the egg surface protein JUNO are essential for sperm-egg binding in mammals [26,27]. In October 2024, Andrea Pauli and Victoria E. Deneke from the Research Institute of Molecular Pathology, Vienna BioCenter, successfully predicted the conserved key trimeric protein Izumo1-Spaca6-Tmem81 involved in vertebrate sperm-egg binding using AlphaFold-Multimer [28]. The rapid progress of artificial intelligence technology and the further integration of computational biology and structural biology are driving the rational design of antigen structures towards precision, offering new strategies for contraceptive vaccine development and enhancing the specificity and safety of antigen recognition.

## 3. The Targets and Mechanisms of Contraceptive Vaccines

### 3.1. Contraceptive Vaccines Targeting Hormones

#### 3.1.1. Gonadotropin-Releasing Hormone (GnRH)

Gonadotropin-releasing hormone (GnRH), a decapeptide synthesized and secreted by the hypothalamus, serves as a crucial hormone regulating reproductive activities. It modulates the synthesis and secretion of luteinizing hormone (LH) and follicle-stimulating hormone (FSH), thereby controlling the production of gonadal steroid hormones and the generation of male and female gametes. Therefore, gonadotropin-releasing hormone (GnRH) represents an ideal dual-functional immunocontraceptive target applicable to both males and females. Anti-GnRH vaccines have been demonstrated to reduce serum testosterone levels in males and suppress testicular development and spermatogenic function [29] while also decreasing progesterone and estradiol levels in females [30].

Due to its weak immunogenicity, GnRH must be coupled with relevant epitope peptides to stimulate B cells or T cells for immune response. Talwar GP et al. immunized male pigs with a GnRH-conjugated T cell epitope peptide, resulting in a sharp decline in testosterone levels, prostate atrophy, and a 100% contraceptive rate [31]. Additionally, researchers have designed a novel conformational G6K-GnRH dimer (G6K-GnRH-tandem-dimer) that exhibits effective immune responses in rats [32]. Its immunogenicity is further enhanced when conjugated with ovalbumin (OVA) [33]. Faruck MO et al. developed self-assembled nanoparticles, PMA-P-GnRH, consisting of GnRH, the universal T helper PADRE, and a polymethacrylic acid (PMA) delivery system [34]. These nanoparticles induce high IgG titers in mice after subcutaneous injection and oral administration. To further enhance immunogenic efficacy, researchers have employed a dual-antigen approach combining GnRH with its upstream regulatory peptide kisspeptin, concurrently conjugated to hepatitis B T-helper peptide sequences. This strategy achieved 100% infertility for 300 days post-primary immunization in pubertal female rats [35].

Early clinical studies revealed that GnRH vaccines completely suppressed testosterone secretion in male subjects, inducing a castration-like physiological state characterized by impotence and body hair loss. Lactating female subjects exhibited amenorrhea [36]. These immunocastration effects have prompted the application of anti-GnRH vaccines for population management in both wild and domesticated animals. GnRH vaccines are not only useful for immune castration but also for treating prostate enlargement and cancer, as well as diseases caused by the excessive secretion of gonadal steroid hormones, such as uterine fibroids, polycystic ovary syndrome, endometriosis, and precocious puberty [37,38]. Although anti-GnRH vaccines provide reliable contraceptive effects, their multiple target organs and the neutralization of GnRH by antibodies leading to the loss of biological activity and reduced sex hormone levels can cause sexual dysfunction and reproductive organ damage, making GnRH vaccines unsuitable for humans [39].

#### 3.1.2. Follicle-Stimulating Hormone (FSH) and Follicle-Stimulating Hormone Receptor (FSHR)

Follicle-stimulating hormone (FSH) is a glycoprotein hormone secreted by the basophilic cells of the anterior pituitary gland, with a molecular weight of approximately 33,000 kD. In males, FSH binds to the FSHR and acts synergistically with androgens to stimulate the synthesis and secretion of androgen-binding proteins and other substances by Sertoli cells, promoting spermatogenesis. Anti-FSH specific antibodies affect spermatogenesis by eliminating the biological activity of endogenous FSH, without affecting the synthesis and secretion of LH; thus, testosterone synthesis remains unaffected. FSH shares the same α-subunit with thyroid-stimulating hormone (TSH) and LH, while only the β-subunit exhibits respective specificity. However, studies have found that male mice with FSH-β gene defects and FSHR inactivation mutations still maintain fertility [40]. In females, follicle-stimulating hormone (FSH) binds to FSH receptors (FSHRs) on ovarian granulosa cells to drive follicular development, estrogen synthesis, and ovulation. Mutations in the FSHR gene (e.g., A189V, I423T, D408Y, rs6165 GG) are associated with primary ovarian failure (POF) or recurrent implantation failure (RIF) in women [41,42,43]. Immunocontraceptive strategies targeting FSH/FSHR inhibit follicular development and subsequent ovulation by blocking the binding of FSH to the FSHR [44].

A novel FSH vaccine based on a tandem repeat of a 13-amino-acid receptor-binding epitope of FSHβ has been reported to severely disrupt steroidogenesis and follicular development in female mice, manifesting as reduced serum estradiol levels, disrupted estrous cycles, arrested follicular development, and decreased litter size [44]. The results of full-length protein vaccine trials for FSHR have not been ideal. Yan P et al. identified three potential B cell epitopes. By synthesizing polypeptides to immunize male rats, they observed reduced testis size, decreased serum testosterone levels, reduced sperm count and quality, and decreased fertility [45]. In another study, male bonnet monkeys immunized with FSHR-57aa underwent fertility testing during the period when antibody titers were highest and stabilizing. The results showed a significant decrease in fertility, and the function of the blood–testis barrier remained intact during the immunization process. Although Sertoli cells were slightly damaged, serum testosterone and estradiol levels remained stable, suggesting that the FSHR-57aa vaccine is a feasible immunization method for male contraception without affecting hormone levels [46]. However, studies have found that the immunization of non-human primates with FSH or FSHR vaccines in male animal experiments can only lead to a reduction in sperm count, rather than completely blocking sperm production. The results of Phase I clinical trials of FSH showed that subjects had low antibody titers, and there was no significant reduction in sperm count [47].

#### 3.1.3. Luteotropic Hormone (LH) and Luteinizing Hormone-Releasing Hormone (LHRH)

Luteotropic hormone (LH) is secreted by the pituitary gland and is also a type of tropic hormone, specifically located in the basophilic cells of the anterior lobe. In the female reproductive system, LH plays a crucial role in promoting the ovulation of fully mature follicles and facilitating the formation of the corpus luteum. In the male reproductive system, LH stimulates the Sertoli cells of the testes to produce testosterone. Suresh et al. immunized Bonnet monkeys with a sheep LH vaccine and found that the generated antibodies effectively bound to LH, resulting in a decrease in testosterone concentration, the inhibition of spermatocyte production, and a reduction in cell count, ultimately leading to azoospermia [48]. The immunization of female primates with LH or its upstream regulator LHRH, has been demonstrated to induce infertility while concurrently disrupting menstrual cycles [49]. The active immunization of heifers with LHRH fused to thioredoxin or OVA resulted in the significant suppression of estrous cyclicity, reduced uterine weight, and produced effects comparable to those observed following ovariectomy [50]. However, LH or LHRH immunization can cause cross-reactions with other pituitary hormones, leading to side effects such as low antibody titers, the atrophy of reproductive organs, and decreased or lost sexual function [51]. Therefore, it has not been widely adopted and subjected to further investigations.

### 3.2. Contraceptive Vaccines Targeting Sperm Antigens

In recent years, significant progress has been made in the study of sperm antigens as targets for immunocontraception. Sperm antigens refer to specific proteins expressed on spermatozoa, such as sperm adhesion molecule 1 (SPAM-1) [52], sperm protein-10 (SP-10) [53], fertilization antigen-1 (FA-1) [54], sperm protein-17 (SP-17) [55], lactate dehydrogenase-C4 (LDH-C4) [56], sperm agglutination antigen 1 (SAGA-1) [57], and YLP-12 peptide [58]. These antigens play crucial roles in the fertilization process of spermatozoa and are of great significance in immunocontraceptive research. By identifying and targeting these antigens, anti-sperm antibodies can be induced in the body, thereby blocking sperm function and leading to the development of novel immunocontraceptive approaches. This provides more options for contraception.

The head region of spermatozoa contains multiple antigens associated with infertility. YLP12, a sequence located in the acrosome region of human spermatozoa, participates in the binding and recognition between spermatozoa and the zona pellucida [59]. The YLP12 peptide can be used to neutralize antibodies. Local injection of the YLP12 peptide into the vagina of female mice during the contraceptive effect phase can neutralize antibodies and restore fertility, achieving a voluntary reversal of contraceptive effects at any time [58].

FA-1 is a specific membrane glycoprotein located in the acrosome of spermatozoa. It plays a role in recognition and adhesion during the process of sperm-egg binding. Anti-FA-1 monoclonal antibodies incubated with spermatozoa in vitro can inhibit sperm capacitation and the acrosome reaction, reducing the binding of spermatozoa to the zona pellucida, and this effect is reversible [54].

Epididymal protease inhibitor (Eppin) is distributed in the acrosome and flagellum of spermatozoa, involved in the binding of spermatozoa to the zona pellucida and the acrosome reaction process, while helping to maintain the structural and functional integrity of the flagellum. Antibodies generated by Eppin recombinant protein immunization can inhibit sperm motility, resulting in 78% of male monkeys becoming infertile. With the decrease in antibody titer, 71% of these male monkeys recover their fertility after immunization [60,61].

Sperm protein-56 (SP-56), located on the outer surface of the plasma membrane of the sperm head, specifically binds to ZP3 oligosaccharides and is a key protein for mammalian sperm–egg recognition [62]. The pre-incubation of unfertilized eggs with recombinant mouse SP-56 reduces the in vitro fertilization rate [63]. Immunizing female mice with recombinant mouse SP-56 five times significantly reduced litter size [64]. However, some studies have found that SP-56 is not essential for mouse fertilization, as homozygous SP-56 knockout males and females show no difference in litter size, and their in vitro fertilization rates are comparable to controls [65].

Izumo, a novel member of the immunoglobulin superfamily (IgSF) of proteins, is localized to the sperm head [66]. To date, four isoforms—Izumo1, Izumo2, Izumo3, and Izumo4—have been identified [67]. In an early study of immunologically infertile men and women, positive reactions to three Izumo peptides were detected exclusively in the sera of infertile females, thereby providing clinical rationale for developing Izumo-based contraceptive vaccines [68]. Xue et al. identified five B cell epitopes within the Izumo sequence; immunization with these epitopes dramatically reduced fertility in female mice and blocked sperm–egg fusion in in vitro fertilization assays [69].

Testicular nuclear autoantigenic sperm protein (tNASP) is a histone chaperone expressed in testicular spermatocytes and spermatids. It participates in cellular proliferation processes such as chromatin assembly and exhibits high autoimmunogenicity [70]. Most men undergoing vasectomy for contraception develop autoantibodies against this protein [71], which may significantly contribute to reduced fertility following vasovasostomy [72]. Consequently, the tNASP antigen may be more suitable for developing female contraceptive vaccines. Notably, only 37.5% of female mice immunized with recombinant mouse tNASP became pregnant and delivered offspring, while the control group reached 87.5%; unfortunately, even after antibody titers gradually declined, the immunized mice remained infertile [73].

LDH-C4 is the first sperm antigen proposed for Phase I clinical trials. Female baboons were tested with a contraceptive vaccine using a synthetic peptide containing B cell epitopes of LDH-C4 and T cell epitopes of tetanus toxin, which significantly and reversibly inhibited fertility [56].

Additionally, the most successful sperm antigen target isolated so far is the sperm membrane protein PH20, also known as SPAM-1 [52]. The active immunization of male and female guinea pigs can cause 100% infertility, and this contraceptive effect is reversible [74].

The antigens on the structure of sperm flagella also constitute significant targets for anti-sperm vaccine research. CatSper, a voltage-dependent calcium channel located in the tail of sperm, demonstrates ideal immunogenicity when administered as a recombinant vaccine in female mice, exerting contraceptive effects [75]. Furthermore, researchers have identified multiple antigens associated with sperm motility through proteomic approaches [76]. For instance, G-protein-coupled receptors (GPCRs) on the sperm membrane are closely linked to sperm capacitation, the acrosome reaction, and motility [77]. It is noteworthy that certain sperm antigens can trigger immune responses when abnormally expressed in tumors, thus emerging as potential targets for immunotherapy [78]. Sperm-associated antigen 6 (SPAG-6), predominantly found in the sperm flagellar structure, plays a crucial role in sperm motility and spermatogenesis, and its absence leads to abnormalities in spermatogenesis and sperm motility disorders [79]. SP-17, a member of the cancer/testis antigen family, is widely distributed in the fibrous sheath of sperm flagella and involved in regulating sperm maturation, capacitation, the acrosome reaction, and the interaction between sperm and the zona pellucida of the egg [55]. The dual functionality of SP-17, which not only plays a role in reproduction but is also highly expressed in tumors, positions it as a potential target for cancer immunotherapy [80]. This dual role makes SP-17 a significant direction in the study of immunocontraception.

Another developmental perspective for sperm antigens stems from anti-sperm antibodies (ASAs) [81]. ASAs were first discovered in the serum of infertile men. Sperm antigens can not only trigger autoimmunity in males but also induce local immunity in the female reproductive tract. The presence of ASAs can affect sperm motility and their ability to bind to eggs, ultimately leading to infertility [82]. Utilizing proteomic techniques, Shetty et al. identified ASAs associated with antibody-mediated infertility from serum samples of both male and female infertility patients, suggesting potential targets for immune contraception [83,84]. In a recent study, researchers extracted antibodies targeting the sperm CD52g antigen from female volunteers with immune infertility and engineered them to capture 99.9% of human sperm. These antibodies have already entered human clinical trials [85]. Currently, the second-generation multivalent antibody structures and other antibody delivery systems, such as vaginal rings and local mRNA applications, are being explored. Furthermore, this antibody, alone or combined with anti-STI measures, is being investigated for use in multipurpose prevention technologies (MPTs) to provide more comprehensive and effective reproductive health protection [9].

The investigation of sperm surface antigen vaccines has presented novel strategies and approaches for contraception. In the advancement of immunocontraceptive vaccines, the identification and targeting of sperm-specific antigens are pivotal. Researchers can now better recognize sperm antigens and devise vaccines with high immunogenicity through techniques such as proteomics and immunoinformatics [86]. Additionally, computer-based simulations of molecular conformations and intermolecular interactions aid in optimizing the design of sperm antigens. A recent study employed molecular dynamics simulations to analyze the conformation of EPPIN, revealing key characteristics of its interaction structure with semen coagulum protein semenogelin-1 (SEMG-1). This allowed for the identification of binding hot spot residues, representing a technological breakthrough in the development of male contraceptives [87].

### 3.3. Contraceptive Vaccines Targeting the Zona Pellucida of Oocytes

The immune contraceptive targets of oocytes primarily involve antigens isolated from the zona pellucida (ZP). The ZP is a glycoprotein structure that surrounds the oocyte, consisting mainly of four glycoproteins: ZP1, ZP2, ZP3, and ZP4 [88]. It plays a crucial role in the process of sperm–egg binding. ZP3, as the primary receptor for sperm, exhibits sperm receptor activity. Its species-specific nature makes ZP3 an ideal target for the development of contraceptive vaccines [89]. Following immunization with the ZP3 vaccine, antibodies occupy the sperm-binding sites on ZP3, preventing sperm from binding to ZP3 and undergoing the acrosome reaction, thus leading to fertilization failure. Additionally, the formation of the ZP3 antigen–antibody complex disrupts the signaling connection between the oocyte and granulosa cells, affecting the normal function of the oocyte and achieving a contraceptive effect.

The earliest application of immunocontraception utilized the zona pellucida antigen derived from porcine zona pellucida 3 (pZP3). The contraceptive vaccine, based on native porcine zona pellucida (pZP), has demonstrated contraceptive efficacy in over eighty animal species in both in vitro and in vivo experiments [90,91]. Studies have revealed that the use of GM-CSF as an adjuvant, combined with the intranasal administration of a mouse ZP3 (mZP3) DNA vaccine, can enhance the humoral immune response and improve contraceptive outcomes [92]. Furthermore, research has explored the application of ZP antigens in other animals. Investigators selected T cell epitopes from dog GnRH and OVA to enhance immunogenicity, constructing a canine ZP3 (cZP3) fused OVA-GnRH-ZP3 (OGZ) recombinant vaccine that significantly reduced fertility rates in mice [93]. Immunocontraception using pZP in wild horses (*Equus caballus*) has been shown to extend the reproductive cycle beyond the normal breeding season, with reversible effects on ovarian function [94]. In non-human primates, such as squirrel monkeys, immunization with pZP3 resulted in a decrease in normal follicles in ovarian histology, although this effect was reversible, suggesting that the selection of appropriate adjuvants can minimize adverse impacts [95].

However, ZP itself possesses certain T cell epitopes, and an excessive immune response against the zona pellucida in the body may lead to autoimmune ovarian diseases, affecting the normal function of the ovaries, such as causing oophoritis and abnormal follicular development, which further impacts female fertility and endocrine balance. Inflammatory infiltration has been observed in histological examinations of mouse ovaries immunized with cZP3 [93]. Due to the potential serious side effects of natural oocyte zona pellucida proteins, many researchers have shifted to using recombinant proteins, DNA immunization, or synthetic peptides as alternatives to natural ZP proteins. Recently, Ghasemian et al. developed several mouse-specific mZP2 and mZP3 contraceptive peptides expressed in *Nicotiana benthamiana*, which exhibit immunogenicity in mice, providing a plant expression system for the development of ZP contraceptive vaccines [96,97]. The immunization of small Indian mongoose (*Herpestes auropunctatus*) with synthetic ZP3 peptides induces a specific antibody response and exerts a contraceptive effect without inducing inflammation or other histological lesions in ovarian tissue [98]. Therefore, in designing target antigens for anti-ZP vaccines, effectively improving vaccine safety and avoiding issues such as oophoritis and follicular atresia can be achieved by identifying and preserving B cell epitopes on the immunogen (such as the 123–126 aa residues of mZP2 [99], the 336–342 aa residues of mZP3 [100], and the 126–130 and 256–260 aa residues of hZP4 [101]), while eliminating T cell epitopes that cause adverse reactions [102]. The application of anti-ZP vaccines in humans requires more rigorous studies to determine their safety and the reversibility of contraceptive effects.

### 3.4. Contraceptive Vaccines Targeting Gamete Outcomes

#### 3.4.1. Human Chorionic Gonadotropin (hCG)

Human chorionic gonadotropin (hCG) is a glycoprotein hormone secreted by placental trophoblast cells. It supports the maintenance of pregnancy in early gestation by promoting the activation of LH receptors [103], playing a critical role in embryo implantation and placenta formation [104]. hCG was initially used to detect female pregnancy, making it an ideal target for immune contraception. Firstly, hCG is almost absent in healthy non-pregnant women and is only produced by the embryo after pregnancy. Therefore, the immune response against hCG exhibits high specificity, causing no interference with other physiological functions of women. Secondly, hCG secretion begins early in gestation and is present in high concentrations in both blood and urine, facilitating detection and serving as a target for immune intervention. Additionally, the structure of hCG is relatively conserved among different individuals, reducing inconsistencies in immune responses due to individual differences.

The hCG vaccine represents the first and only contraceptive vaccine to have successfully passed Phase II clinical trials. Currently, there are two main types of hCG vaccines: one uses the entire β-subunit of hCG (β-hCG) as the antigen, while the other employs the β-hCG carboxy-terminal peptide (β-hCG-CTP) as the vaccine antigen. Studies have shown that both hCG and β-hCG-CTD exhibit immunogenicity, capable of inducing high-titer antibodies in the body. In clinical trials, when anti-hCG antibody titers reach a certain level (such as above 50 ng/mL), they can effectively prevent pregnancy without significantly affecting female ovulation function and menstrual cycle. Fertility can be restored when antibody titers drop below 35 ng/mL. No side effects were observed in Phase I and Phase II trials [105,106]. However, the vaccine still has limitations, as only 60–80% of women can maintain high-titer protective antibodies [107]. To enhance the immunogenicity and efficacy of the vaccine, researchers have utilized various carriers such as diphtheria toxin and tetanus toxin to induce higher titer antibodies. Additionally, the incorporation of three promiscuous pathogen-derived Th peptides has been found to increase the immunogenicity of the hCG vaccine [108].

#### 3.4.2. Leukemia Inhibitory Factor (LIF)

Leukemia inhibitory factor (LIF), a member of the interleukin-6 family, stands as a crucial cytokine in immune regulation at the maternal–fetal interface [109]. It plays a pivotal role in embryonic development and the successful implantation of blastocysts [110]. Functional deficiencies in the LIF or its receptor can lead to implantation failure [111,112]. Clinically, fertile women have been found to have higher LIF immunostaining upon biopsies compared to infertile women [113]. Moreover, mutations in the LIF gene among women with unexplained infertility and endometriosis negatively impact in vitro fertilization (IVF) outcomes [114]. In animal studies, the administration of a high-affinity LIF antagonist has been shown to completely block the implantation process in mice embryos. Mutations in the LIF gene can result in infertility among mice [115,116]. Immune intervention targeting the LIF offers the potential to specifically block the pregnancy process while minimally affecting other physiological functions. Therefore, the LIF is considered a potential target for immunocontraception. Lemons et al. selected five peptide segments spanning the ligand–receptor binding region on LIF and its receptor, LIF-R, to develop a vaccine. The immunization of female mice induced long-lasting circulating antibodies and local antibody responses in the reproductive tract. These antibodies persisted in the mice for up to 11 months after the final immunization, significantly inhibiting fertility. Notably, the LIF-R peptide vaccine demonstrated superior contraceptive efficacy [117]. Furthermore, the inhibition of the LIF can be utilized not only for contraception but also in combination with other microbial inhibitors to prevent sexually transmitted infections, providing dual protection [115].

## 4. Advantages of Contraceptive Vaccines

### 4.1. High Efficiency and Long-Lasting Effect

Due to the core mechanism of immunological contraception, which involves triggering the body’s own immune defense mechanisms through precisely designed antigenic epitopes to prevent unplanned pregnancies, contraceptive vaccines possess the same specific and durable immune protection as other vaccine drugs. Designed to target specific reproductive system components, contraceptive vaccines significantly enhance the specificity and effectiveness of contraception [118].

During the development of contraceptive vaccines, researchers have identified key target molecules involved in pregnancy, such as hCG and the LIF [118]. Additionally, multi-epitope recombinant proteins, which can effectively stimulate the immune system to inhibit reproductive function, are being considered as candidates for contraceptive vaccines [119]. Furthermore, contraceptive peptides produced by plants have demonstrated good immunogenicity and contraceptive effects, offering the possibility of developing oral contraceptive vaccines [97]. By developing multi-epitope vaccines targeting these molecules, the immunogenicity and contraceptive efficacy of the vaccines can be improved [120]. This high efficiency not only reduces the risk of unplanned pregnancies but also provides a better option for those with long-term contraceptive needs, such as women who have completed their family planning.

Similar to other types of vaccines, contraceptive vaccines offer the advantage of long-lasting effects. This longevity arises from the immune memory effect induced by the vaccine, which stimulates the production of specific antibodies (such as anti-hCG antibodies) that continuously block key targets in the reproductive process, providing durable contraceptive effects [119]. Compared to traditional contraceptive methods, contraceptive vaccines can offer months or even years of contraceptive protection with a single vaccination. This not only improves the success rate of contraception but also reduces the hassle of frequent use of other contraceptive methods, greatly enhancing the convenience and compliance of contraception and minimizing the risk of unplanned pregnancies due to negligence.

### 4.2. Non-Invasiveness and Convenience

Traditional contraceptive methods, such as the placement of an IUD, require invasive surgical procedures to insert the device into the uterine cavity. These invasive operations carry a certain risk of infection and may cause discomfort to the user during insertion and removal. Sterilization surgeries, like female tubal ligation or male vasectomy, involve even more invasive procedures with relatively greater physical trauma and require a period of postoperative recovery. In stark contrast, the administration of contraceptive vaccines is similar to that of regular vaccines, typically involving intramuscular or subcutaneous injection. This simple and minimally invasive approach significantly reduces the risks associated with invasive procedures, such as infection, and greatly enhances user acceptance.

Contraceptive vaccines offer remarkable convenience in terms of usage. Traditional contraceptive methods, including contraceptive pills and devices, may require long-term, stable administration or periodic replacement. However, contraceptive vaccines achieve their effect through a simple injection. This method not only simplifies the user’s operation but also reduces the risk of contraceptive failure due to operational errors [121]. Additionally, unlike condoms, which require preparation before each sexual activity, contraceptive vaccines eliminate the need for such preparations, further enhancing their convenience and user satisfaction, and thus better achieving contraceptive goals [122].

### 4.3. Reversibility and Safety

Unlike some permanent contraceptive methods such as sterilization, the effect of contraceptive vaccines is reversible. Once vaccination is stopped, fertility gradually recovers. This provides more options and flexibility for women who temporarily do not want to have children but have plans for future fertility [123].

Contraceptive vaccines have significant advantages in terms of safety compared to traditional contraceptive methods like hormonal contraceptives. Steroidal contraceptives achieve contraception by regulating hormonal levels in the body. However, this hormonal manipulation often triggers a range of side effects, including menstrual cycle disruptions. Additionally, contraceptives may increase the risk of depression and suicidal attempts. Users of combined oral contraceptives (COCs) have a higher risk of depression compared to controls [124], and adolescents (aged 15–19) using contraceptives are at an elevated risk of depression [125]. A study examining national registry data from Sweden found that oral contraceptive users had an increased risk of suicidal behavior (attempted suicide or suicide death) after one month of use, but only progesterone-only pill (POP) users maintained a higher risk after one year of use [126]. Conversely, contraceptive vaccines primarily achieve contraception by inducing an immune response in the body, without involving hormonal manipulation. Therefore, they can effectively avoid the hormone-related side effects associated with steroidal contraceptives [127]. The immune response to contraceptive vaccines is relatively mild. Although some minor local reactions, such as redness and pain at the injection site, may occur during the initial vaccination period, these reactions typically resolve spontaneously within a short time and do not have long-term impacts on physical health.

### 4.4. Promoting Gender Equality

Traditional contraceptive methods often require women to bear more responsibility. Globally, among married couples or sexual partners using contraceptive measures, 79.1% rely on female-specific contraceptive methods, while male contraceptive methods account for only 20.8% [128]. However, with the clinical trials of injectable male contraceptives [129] and non-hormonal male contraceptives [130,131], the topic of contraceptive responsibility in both genders has attracted public attention. That is, in a relationship, contraception is not solely the responsibility of women; men should also bear corresponding obligations. A recent study has found a significant positive correlation between attitudes towards gender equality and men’s willingness to use contraceptive measures [132]. Male contraceptive vaccines targeting sperm antigens, such as Eppin and CD52g, provide new possibilities for male contraception, promoting equality in gender roles [133]. Furthermore, the widespread use of contraceptive vaccines can reduce the occurrence of unwanted pregnancies, thereby reducing the social and economic pressures faced by women due to unexpected pregnancies and further improving women’s quality of life and social status [134]. Contraceptive vaccines have positive implications in promoting gender equality.

## 5. Limitations of Contraceptive Vaccines

Although contraceptive vaccines demonstrate significant advantages in terms of high efficacy, long duration, and safety, their application still faces several limitations (Table 1).

### 5.1. Individual Variation in Immune Response

Similar to other types of vaccines, the immunogenicity of contraceptive vaccines is influenced by factors such as genetic background, age, and health status, leading to considerable individual variation. Certain populations may exhibit reduced antigen-presentation efficiency due to HLA genotype variations [135], resulting in insufficient antibody titers or shorter duration of immune protection. Age-related immunosenescence may also weaken vaccine-induced immune memory [136,137], thereby compromising the long-term contraceptive efficacy. Furthermore, certain chronic disorders (e.g., diabetes) [138] or the prolonged use of medications (e.g., immunosuppressants) [139] may suppress the immunogenicity of the vaccine, further diminishing its effectiveness.

### 5.2. Potential Autoimmune Risks and Controversies Regarding Safety and Reversibility

Contraceptive vaccines require targets that exhibit both reproductive specificity and immunogenicity, which may trigger autoimmune responses. Immunization against the FSHR has been shown to induce mild damage to Sertoli cells in bonnet monkeys [46]. ZP inherently contains T cell epitopes, and an excessive immune response to ZP may lead to oophoritis and follicular atresia [102]. Immunization against LH may result in cross-reactivity with other pituitary hormones, causing adverse effects such as atrophy of reproductive organs and impaired or lost sexual function [51]. Due to their chemical and biological stability, DNA vaccines have also been explored for contraceptive vaccine development [92]. However, caution is warranted, as DNA vaccines may enhance Th1-mediated immune responses, leading to the secretion of cytokines that negatively affect gamete and embryo function [140].

At present, the reversibility of contraceptive vaccines remains uncertain. Although antibody levels theoretically decline over time, certain vaccines may induce robust immune responses (e.g., persistent memory B cell responses), resulting in contraceptive effects that far exceed the anticipated duration and even lead to irreversible gonadal suppression [141]. Vaccines targeting gonadotropin-releasing hormone (GnRH) represent a major research focus in immunocastration, with systematic studies and applications conducted in male rats [142], Tibetan sheep [143], sheep [144], and pigs [145]. It must be emphasized that the core principle of immunocontraception lies in ensuring reversibility—contraceptive effects should completely resolve upon antibody clearance without causing permanent damage to reproductive tissues. In contrast, immunocastration irreversibly abolishes gonadal steroidogenesis, leading to permanent infertility and hormone deprivation effects. Although both approaches are immunologically based, they differ fundamentally in mechanism and outcome. Reversibility remains the ethical cornerstone and prerequisite for the application of immunocontraception.

### 5.3. Population Restrictions for Contraceptive Vaccine Use

#### 5.3.1. Individuals with Multiple Sexual Partners

The contraceptive vaccine inhibits fertilization by inducing an immune response, but it does not provide protection against STIs [120,146]. Vaccination may inadvertently increase health risks due to reduced STI protection awareness. Therefore, it is unsuitable for individuals with multiple sexual partners or those at high risk of STIs.

#### 5.3.2. Individuals Planning Pregnancy in the near Term

Similar to other vaccines, the metabolism of antibodies and the resolution of immune memory exhibit interindividual variability [147]. Consequently, the precise duration of contraceptive efficacy cannot be reliably predicted. Notably, early clinical trials observed amenorrhea in lactating female subjects receiving GnRH vaccines, raising additional concerns regarding potential detrimental effects on bone metabolism and infant development [36].

#### 5.3.3. Patients with Immunodeficiency or Autoimmune Diseases

As discussed in Section 5.1 “Interindividual Variability in Immune Response”, individuals with immune dysfunction or those subjected to immunosuppressive therapy may fail to mount an effective immune response. Additionally, vaccination may trigger or exacerbate autoimmune reactions.

**Table 1:** Advantages and limitations of contraceptive vaccines.

Advantages		
Item	Key Point	Additional Details
Specificity	Triggering protective immunity via precisely designed epitopes	Antigen-specific immune response
Long-lasting Effect	Single-administration protection for months to years	Immunological memory effect
High Efficiency	Enhanced immunogenicity through multi-epitope combinatorial vaccines	Epitope diversity/B cell receptor diversity
Non-invasiveness	Injection delivery eliminating surgical trauma	Postoperative sequelae of IUD/sterilization
Adherence	Streamlined protocols with low implementation costs	No daily dosing/pre-coital preparation required
Convenience	Reduced failure risk from operational errors	
Reversibility	Fertility recovery upon discontinuation	Uncertainty in vasectomy reversal efficacy
Safety	Minimal hormonal interference preventing depression/suicide risks	Distinct from oral contraceptives
Safety	Transient local reactions (redness/pain) without long-term sequelae	Post-procedural complications of IUD/sterilization
Gender Equality	Proactive male vaccination engagement	Societal implication: Shared responsibility
	Alleviated socioeconomic burdens from unintended pregnancies on women	Gender equality: Burden redistribution
**Limitations**		
**Item**	**Key Point**	**Additional Details**
Individual Variation in Immune Response	HLA genotype variations impair antibody titers	Genetic background
	Age-related immunosenescence compromises immune memory	Immunosenescence and reducing memory B cell generation
	Chronic diseases/immunosuppressants diminish vaccine efficacy	Diabetic states/glucocorticoids suppress Tfh cell differentiation
Potential Autoimmune Risks	Reproductive-specific targets risk triggering autoimmunity	FSHR antibodies target Sertoli cells
	Intrinsic T cell epitopes in antigens	ZP antigens harbor dominant Th1 epitopes risking oophoritis
	Cascade interference of hormonal axes	LH vaccines cross-inhibit TSH/ACTH secretion
Unpredictability of Reversibility	Longevity of memory B cell pools	Bone marrow-resident plasma cells sustain antibody production > 2 years
	Irreversible gonadal suppression	Early GnRH vaccine trials demonstrated castration-like effects
Population Restrictions for Contraceptive Vaccine Use	Individuals with multiple sexual partners	No protection against STIs
	Individuals planning pregnancy in the near term	Individual variability in antibody decay and immune memory waning
	Patients with immunodeficiency or autoimmune diseases	Immune hyporesponsiveness or autoimmune induction

## 6. Conclusions

Significant progress has been made in recent years in the research of contraceptive vaccines in reproductive health. Despite facing numerous challenges, such as vaccine specificity, long-term effectiveness, and safety issues, researchers have gradually overcome many technical obstacles through the continuous exploration of novel antigens and immunostimulants. Currently, the development of immunocontraceptive vaccines has demonstrated promising results in various animal models. Future research needs to further optimize the immunogenicity and safety of the vaccines to achieve broader applications. In summary, with the continuous advancement of science and technology, immunocontraceptive vaccines are expected to become an effective and sustainable contraceptive method, offering new solutions for global population control and animal population management.

## Abbreviations

The following abbreviations are used in this manuscript:

hCG	Human chorionic gonadotropin
GnRH	Gonadotropin-releasing hormone
LH	Luteinizing hormone
FSH	Follicle-stimulating hormone
OVA	Ovalbumin
PMA	Polymethacrylic acid
FSHR	Follicle-stimulating hormone receptor
TSH	Thyroid-stimulating hormone
SPAM-1	Sperm adhesion molecule 1
SP-10	Sperm protein-10
FA-1	Fertilization antigen-1
LDH-C4	Lactate dehydrogenase-C4
SAGA-1	Sperm agglutination antigen 1
SP-17	Sperm protein-17
Eppin	Epididymal protease inhibitor
GPCRs	G-protein-coupled receptors
SPAG6	Sperm-associated antigen 6
ASA	Anti-sperm antibody
STIs	Sexually transmitted infections
IUD	Intrauterine device
MPTs	Multipurpose prevention technologies
SEMG-1	Semen coagulum protein semenogelin-1
ZP	Zona pellucida
pZP	Porcine zona pellucida
LIF	Leukemia inhibitory factor
IVF	In vitro fertilization
COCs	Combined oral contraceptives
POP	Progesterone-only pill
LHRH	Luteinizing hormone-releasing hormone
POF	Primary ovarian failure
RIF	Recurrent implantation failure
tNASP	Testicular nuclear autoantigenic sperm protein
SP-56	Sperm protein-56
CV	Contraceptive vaccine
